# Systemically Administered IgG Anti-Toxin Antibodies Protect the Colonic Mucosa during Infection with *Clostridium difficile* in the Piglet Model

**DOI:** 10.1371/journal.pone.0111075

**Published:** 2014-10-27

**Authors:** Ocean R. Cohen, Jennifer A. Steele, Quanshun Zhang, Diane J. Schmidt, Yuankai Wang, Philip E. S. Hamel, Gillian Beamer, Bingling Xu, Saul Tzipori

**Affiliations:** Department of Infectious Disease and Global Health, Tufts Cummings School of Veterinary Medicine, North Grafton, Massachusetts, United States of America; Cornell University, United States of America

## Abstract

The use of anti-toxin human monoclonal antibodies (HMab) as treatment for *C. difficile* infection has been investigated in animal models and human clinical trials as an alternative to or in combination with traditional antibiotic therapy. While HMab therapy appears to be a promising option, how systemically administered IgG antibodies protect the colonic mucosa during *Clostridium difficile* infection is unknown. Using the gnotobiotic piglet model of *Clostridium difficile* infection, we administered a mixture of anti-TcdA and anti-TcdB HMabs systemically to piglets infected with either pathogenic or non-pathogenic *C. difficile* strains. The HMabs were present throughout the small and large intestinal tissue of both groups, but significant HMabs were present in the lumen of the large intestines only in the pathogenic strain-infected group. Similarly, HMabs measured in the large intestine over a period of 2–4 days following antibody administration were not significantly different over time in the gut mucosa among the groups, but concentrations in the lumen of the large intestine were again consistently higher in the pathogenic strain-infected group. These results indicate that systemically administered HMab IgG reaches the gut mucosa during the course of CDI, protecting the host against systemic intoxication, and that leakage through the damaged colon likely protects the mucosa from further damage, allowing initiation of repair and recovery.

## Introduction

*Clostridium difficile* is an anaerobic, spore-forming, gram-positive bacterium, and the most frequent cause of antibiotic-associated diarrhea in humans. Like other clostridia, *C. difficile* is a toxin-producer, and pathogenic effects are due primarily to the two large clostridial glucosylating toxins, toxin A (TcdA) and toxin B (TcdB). These toxins are enterotoxic and cause increased mucosal permeability by inducing intestinal epithelial cell damage [1]. Both TcdA and TcdB consist of three major domains: the N-terminal catalytic domain, the central translocation domain, and the C-terminal receptor binding domain [2]. By inactivating Rho family GTPases in the gut epithelial cells, the toxins disrupt cell signaling, which leads to disruption of the tight junctions, cytoskeletal degradation, cell rounding, and cell death [1], [2].

The symptoms of *C. difficile* infection (CDI) in humans range from asymptomatic carriage to severe pseudomembranous colitis, toxic megacolon, and death [3]. The historic gold standard treatment for CDI is administration of metronidazole or vancomycin and discontinuation of the previously administered broad-spectrum antibiotics [4]. Treatment failures as well as frequent recurrence in antibiotic-treated patients has led to the search for more effective treatment options, which currently include novel antimicrobials, fecal transplantation, probiotic supplementation, and anti-toxin antibodies [4], [5]. In fact, human monoclonal antibodies (HMab) against TcdA and/or TcdB effectively treat CDI in the hamster model [6] as well as in the piglet model in our laboratory [7], and, in combination with either metronidazole or vancomycin, significantly reduce CDI recurrence rate in humans [5].

These anti-toxin antibodies are administered systemically by intravenous or intraperitoneal injection in the animal models and intravenously in human patients, but little is known as to how these systemically administered IgG antibodies protect the colonic mucosa during CDI. Suggested mechanisms of action for systemically administered HMabs are that they either transfer to the gut lumen via a leaky mucosal barrier [8] or they may be actively transported by an IgG neonatal Fc receptor [9], [10]. Knowing that the *C. difficile* toxins increase intestinal mucosal permeability by disrupting tight junctions, our hypothesis is that the antibodies leak from the mucosal blood capillaries into the lumen through mucosa damaged by CDI. Thus, we expected that intestinal mucosal damage induced by pathogenic *C. difficile* would be associated with greater concentrations of systemically administered HMab in the gut lumen. We investigated this in groups of piglets that were inoculated with either pathogenic (UK6) or non-pathogenic (CD37) strains of *C. difficile* to measure the presence of the HMabs at different sites of the gut mucosa and in the gut lumen of both groups.

## Methods

### Monoclonal anti-toxin antibody preparation

The human monoclonal anti-TcdA (CDA1) and anti-TcdB (CDB1) antibodies used in this study were developed by Massachusetts Biologic Laboratories and Medarex, Inc. [6], and were provided for this study and currently licensed by Merck, Inc. These antibodies have already been used in the hamster model [6], the piglet model [7], and in clinical trials in humans [11], [12]. Both CDA1 and CDB1 are IgG1κ antibodies and bind the receptor-binding domain of TcdA and TcdB, respectively [6]. CDA1 and CDB1 were administered to piglets at a dose of 10 mg/kg suspended in sterile PBS via intraperitoneal injection [11], [12]. The dose used in piglets was based on that given to humans in clinical trials, as well as the protective dose in piglets in past experiments in our laboratory [7].

### Animals and inoculation

Piglets were derived via Cesarean section from a conventional sow (Parson's Farm) and maintained in sterile isolators for the duration of the experiment, as we have previously described [13]. A total of 23 gnotobiotic piglets were randomly divided into 3 groups: 2 piglets were not infected and treated with CDA1 and CDB1 to determine if these anti-toxin IgGs crossed from the systemic circulation to the gut lumen in the absence of bacterial colonization and to monitor for adverse events associated with CDA1 and CDB1; 9 piglets were orally inoculated with 10^8^ vegetative cells of non-pathogenic *C. difficile* strain CD37 and treated with CDA1 and CDB1; and 12 piglets were orally inoculated with 10^8^ spores of pathogenic *C. difficile* strain UK6 [14] and treated with CDA1 and CDB1 (Table 1). Randomization was completed immediately after birth by the animal care technicians who were unaware of treatments animals in each group would receive. Additionally, data from 4 UK6-inoculated piglets which received an irrelevant anti-Stx2 HMab from previous experiments in our laboratory [7] were used for antibody controls (Table 1). All animals were cared for according to ethical procedures to reduce pain and suffering by protocol approved by the Institutional Animal Care and Use Committee of Tufts University (protocol #G2013-83).

**Table 1:** Clinical signs and intestinal lesions in piglets inoculated with *C. difficile* and treated with anti-toxin antibodies.

C. difficile strain	clinical signs	mortality	gross intestinal lesions	histopathologic lesions	C. difficile range (cfu/ml)
**Pathogenic UK6, treated with CDA1 and CDB1 (n = 12)**	mild-moderate diarrhea, transient anorexia	none	mild-moderate mesocolonic edema, rectal dilatation	mild-moderate mesocolonic and submucosal edema	10^5^–10^10^
**Pathogenic UK6, treated with irrelevant control** A **(n = 4)**	moderate-severe diarrhea, anorexia, lethargy	2/4	moderate-severe mesocolonic edema, pseudomembranous colitis, mucosal congestion	moderate-severe mesocolonic and submucosal edema, marked neutrophilic infiltration, mucosal erosion and ulceration, luminal psueudomembranes	10^6^–10^10^
**Nonpathogenic CD37 treated with CDA1 and CDB1 (n = 9)**	none	none	none	none or minimal mesocolonic edema	none
**Noninfected and treated with CDA1 and CDB1 (n = 2)**	none	none	none	none or minimal mesocolonic edema	n/a

Notes: CDA1  =  anti-toxin A human monoclonal antibody, CDB1 =  anti-toxin B human monoclonal antibody.

A control piglets were treated with irrelevant anti-Stx2 human monoclonal antibody [7].

At 48 hours after inoculation, the piglets receiving the pathogenic strain UK6 developed diarrhea, and at that point a mixture of 10 mg/kg of CDA1 and 10 mg/kg CDB1 was administered by intraperitoneal injection to all piglets in each group. Piglets were observed at least 4 times daily for signs of CDI including diarrhea, perianal inflammation, weakness, lethargy, and anorexia. Piglets were euthanized at a predetermined time between 2 and 4 days post treatment with CDA1 and CDB1 (Table 1). Blood samples were collected immediately prior to euthanasia. Gut luminal contents and tissue samples from the duodenum, jejunum, ileum, cecum, spiral colon, and rectum were harvested after euthanasia.

### Human monoclonal antibody quantification

Concentrations of CDA1 and CDB1 in the serum, gastrointestinal contents, and gastrointestinal tissues were measured by ELISA. Prior to use, gastrointestinal contents were diluted at a 1∶10 ratio using sterile PBS and centrifuged at 9000 rpm for 3 minutes. The same technique was used for the 6 sections of gastrointestinal tissues prior to homogenization and centrifugation. Serum was not diluted in PBS and was used at body concentrations. High binding capacity 96-well plates were coated with 0.5µg/ml of rTcdA or rTcdB [15] using PBSN coating buffer and left covered overnight at 4°C. Plates were washed using a PBST buffer before being blocked with 0.1% BSA-PBST for 30 minutes at room temperature. Plates were washed, and two-fold serial dilutions of either CDA1 or CDB1 were used as standards beginning at 100 ng/ml and added at 100µl/well followed by the addition of the collected samples and positive and negative controls at 100µl/well and incubated at room temperature for 1 hour. Plates were washed, and donkey anti-human IgG-HRP conjugate was used as the secondary antibody. Plates were developed and the OD_450_ was measured. Mean antibody concentrations were statistically compared using the independent samples t-test for comparisons between the UK6 and CD37-infected groups and using ANOVA for comparisons among the 3 daily time points. Samples from all animals were included in the analysis.

### Histopathology

Samples collected for histological examination were fixed in 10% neutral buffered formalin for routine processing, sectioning, and staining with hematoxylin and eosin at TCSVM Histopathology Service Laboratory. We sampled each of the 6 sections of the gastrointestinal tract as well as the heart, lungs, liver, spleen, and kidney. A board certified veterinary pathologist (GB) assessed the tissues for lesions characteristic of CDI including edema, ulceration, erosion, and suppurative (neutrophilic) inflammation.

## Results

### CDA1 and CDB1 protect against mortality, morbidity, and severity of intestinal lesions

All piglets inoculated with pathogenic UK6 developed typical signs of CDI with diarrhea and anorexia, 2 days post-inoculation, at which point they were treated with CDA1 and CDB1 HMabs. Piglets treated with a mixture of CDA1 and CDB1 two days after inoculation with the UK6 strain of *C. difficile* were protected from development of severe clinical signs of CDI as well as severe injury to the colonic mucosa, systemic intoxication, and mortality, compared with the UK6-infected, irrelevant anti-Stx2 HMab treated controls (Table 1), as well as untreated UK6-infected piglets, which we have previously described [7], [13]. The UK6-infected piglets that received anti-Stx2 HMab instead of CDA1 and CDB1 developed typical CDI disease with moderate to severe pseudomembranous colitis, and half of the piglets developed fatal disease with systemic lesions (Table 1). *C. difficile* cultures for UK6 were performed on large intestinal contents collected during necropsy, but were not significantly different between groups treated with CDA1 and CDB1 vs anti-Stx2 HMabs (Table 1). *C. difficile* cultures for CD37 failed to confirm growth of this strain from the infected group of piglets, likely due to failure of the strain to sporulate and exposure of samples to oxygen during processing (Table 1). Diarrhea improved or resolved in all 12 CDA1 and CDB1 treated piglets following administration (Table 1). On histopathologic examination, lesion severity in UK6-infected, CDA1 and CDB1 treated piglets was reduced to mild-moderate mesocolonic or submucosal edema in the spiral colon and rectum of some of the piglets and there was no evidence of pseudomembranous colitis (Table 1). As expected, the piglets inoculated with the non-pathogenic strain CD37, as well as the uninfected controls treated with CDA1 and CDB1, developed no clinical signs of illness and had either no observed intestinal lesions or minimal mesocolonic edema (Table 1).

### CDA1 and CDB1 presence in mucosal tissues and gut contents after systemic administration

**Figure 1:**
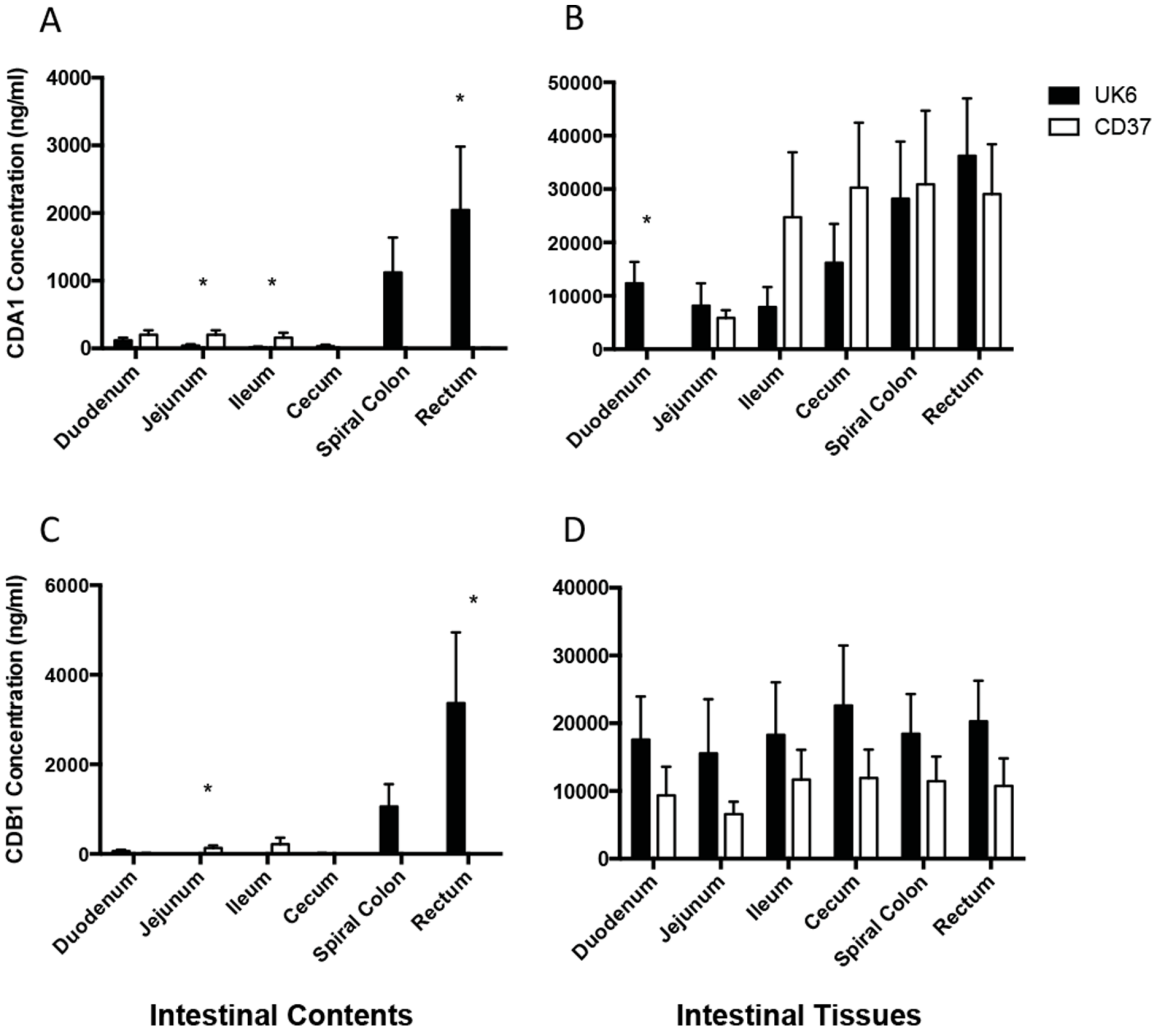
Concentrations of human monoclonal antibodies CDA1 and CDB1 in the intestinal contents and tissues. Gnotobiotic piglets were inoculated with either pathogenic UK6 or nonpathogenic CD37 and treated systemically with human monoclonal anti-toxin antibodies against TcdA and TcdB (CDA1 and CDB1) 2 days following inoculation. **A**. CDA1 in intestinal contents **B**. CDA1 in intestinal tissues **C**. CDB1 in intestinal contents **D**. CDB1 in intestinal tissues. * p<0.05 between groups using the independent samples t-test. Bars represent the mean with standard error.

CDA1 and CDB1 were measureable in all mucosal tissues evaluated, including the small intestine (Figure 1, B and D). Both HMabs were present in the gut contents in appreciable amounts only in the large intestine of UK6-infected piglets and at significantly higher concentration in the rectum, where most water absorption from the contents has been completed (Figure 1, A and C). Minimal amounts of both HMabs were measured in the small intestinal contents of CD37 or UK6-infected piglets (Figure 1, A and C), which we attribute to the lack of any mucosal lesions in the small intestine in either group. HMab concentrations in the mucosal tissues were ∼10 fold greater than in the lumen of the large intestine, with maximal concentrations of nearly 50,000 ng/ml in the large intestinal mucosa (Figure 1, B and D). Figure 1 also reflects the relative distribution of the two HMabs throughout the gut tissues with no trend for significant differences in antibody concentration between CD37 and UK6-infected piglets (Figure 1, B and D). While the focus of analysis was on differences between the two CDI groups, the two uninfected piglets had the same pattern of HMab distribution as the CD37-infected group (data not shown).

### CDA1 and CDB1 persist in intestinal mucosal tissues and contents

**Figure 2:**
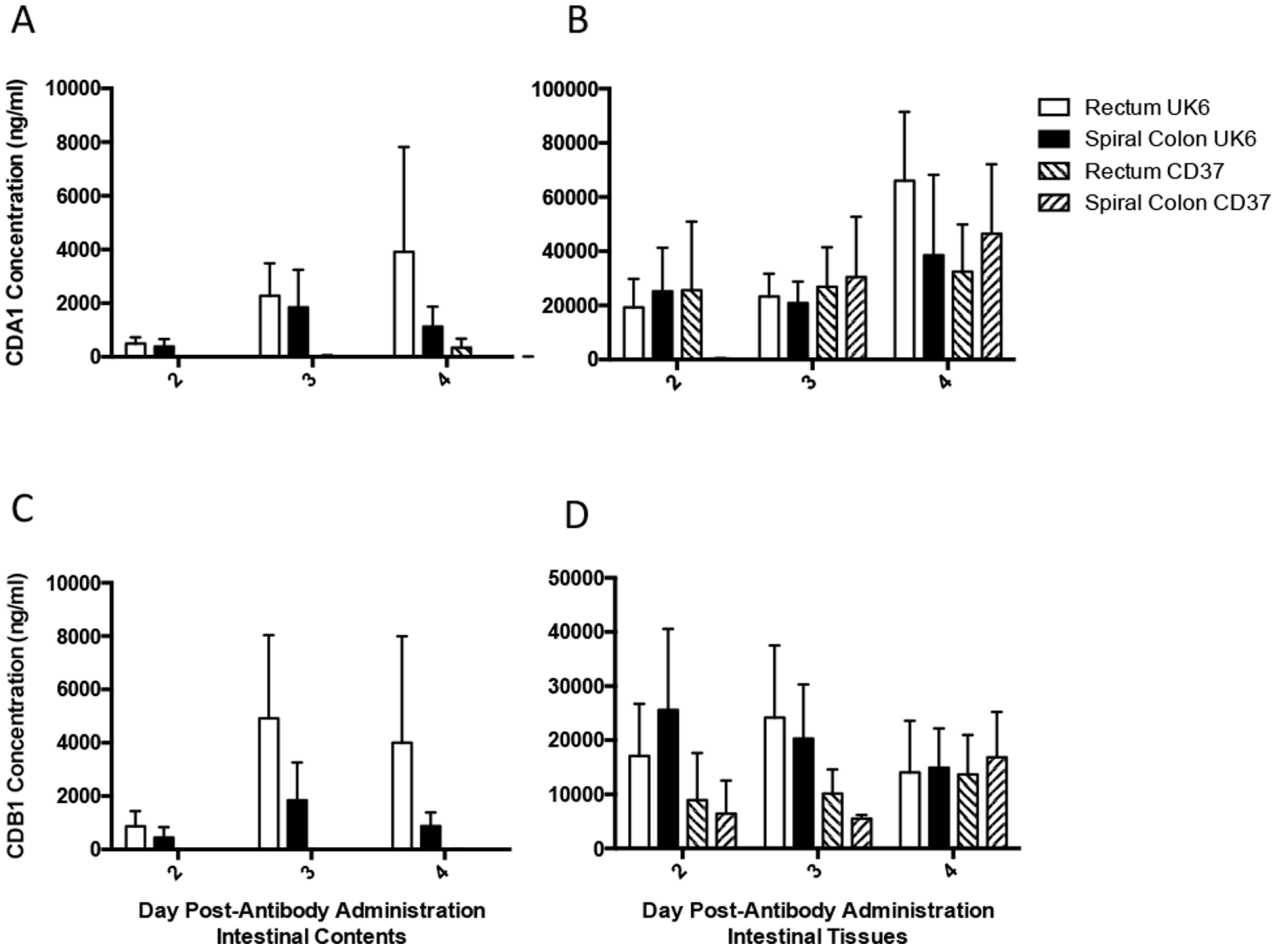
Concentrations of human monoclonal antibodies CDA1 and CDB1 in the large intestinal contents and tissues over time following systemic administration in piglets inoculated with *C. difficile*. Gnotobiotic piglets were inoculated with pathogenic *C. difficile* strain UK6 and then treated once systemically with human monoclonal anti-toxin antibodies against TcdA and TcdB (CDA1 and CDB1) 2 days following inoculation, at the onset of clinical signs. Piglets were euthanized and tissues collected 2, 3, or 4 days following antibody administration. **A.** CDA1 in large intestinal contents **B.** CDA1 in large intestinal tissues **C.** CDB1 in large intestinal contents **D.** CDB1 in large intestinal tissues. Bars represent the mean with standard error.

CDA1 and CDB1 were measured in the gut contents and mucosal tissues in groups of piglets infected with UK6 or CD37 and euthanized at 2, 3, and 4 day time points after HMab administration to evaluate the concentration over time. While we found no significant differences in HMab concentrations over this time period in the mucosal tissues or contents (Figure 2), we noticed a trend for increased concentration in the large intestinal contents on days 3 or 4 in the UK6-infected group (Figure 2, A and C). Comparison of CDA1 and CDB1 concentrations in the tissues and contents between the CD37 and UK6-infected groups shows a trend of elevated concentrations in contents of the spiral colon and rectum of the UK6-infected group. The difference was not statistically significant in this analysis, likely due to smaller group sizes when piglets were stratified by day of sampling (Figure 2, A and C).

## Discussion

In this study we show an association between colonic injury due to CDI and the presence of IgG in the gut lumen, as compared with normal intestinal mucosa. The anti-toxin HMabs used in these experiments have been shown to be effective in modifying the clinical outcome of CDI in the hamster and gnotobiotic piglet models, and in clinical trials with human patients [5], [6], [11], [12]. However, it is unclear how systemically administered IgG antibodies are able to provide protection against bacterial toxins liberated in the lumen of the large intestine. We believe that the presence of systemically administered CDA1 and CDB1 HMabs in the gut contents is likely attributed to leakage through the mucosal injury inflicted by the bacteria and/or toxins to the protective mucosal surface, therefore confirming the hypothesis that damage to the gut mucosa facilitates antibody leakage into the gut lumen.

While we cannot entirely rule-out the contribution of active transport of IgG by the neonatal Fc receptor [9], we would not expect the marked difference in HMab concentrations in the gut contents between groups infected with pathogenic and non-pathogenic bacterial strains, as we have shown in these experiments. Antibodies leaking into the gut lumen neutralized the bacterial toxins, thus protecting the mucosa from further damage by the pathogenic strain. Furthermore, the presence of HMab IgG antibody in the mucosal vasculature intercepts and neutralizes toxin uptake from the lumen thus preventing the occurrence of systemic intoxication, which can be observed in patients and consistently in piglets with CDI [16]. Perhaps if HMabs were administered after piglets began to develop more severe clinical signs of CDI after inoculation, a more likely event for human patients, the degree of antibody leakage into the lumen would be even greater. While the contribution of toxin production to *C. difficile* colonization of the large bowel is unknown, Shiga toxin was shown to facilitate the colonic colonization of *E. coli* O157 [17], and it is possible that the same may apply to TcdA and TcdB. It was also difficult to accurately quantify *C. difficile* vegetative cells collected from intestinal contents, as they are quickly destroyed upon contact with oxygen, thus counts likely reflect predominantly spores in the contents samples. As reflected above in the results, we failed to recover CD37 from the intestinal contents of piglets infected with the strain, which may also be due to poor sporulation this strain and exposure of the samples to oxygen. While the degree of colonization of strain CD37 in this group of animals cannot be confirmed, the group still represents a non-pathogenic, non-toxin control with which to compare the clinical and laboratory findings from the group infected with the pathogenic UK6 strain.

We found no significant difference in concentration of either antibody over time between the UK6 and CD37 groups, as seen in Figure 2 depicting the concentration of antibodies in the large intestinal mucosa and contents of the piglets; only in the gut lumen. There was no significant increase in the amount of leakage of antibodies into the lumen over the three days they were quantified, indicating that a single systemic administration of the two HMabs results in greater concentrations in the large intestinal mucosa for at least 4 days, with a trend of increased concentrations in the large intestinal lumen after 2 days in the UK6-infected piglets.

In summary, our investigation has shown that 1) these IgG HMabs were present throughout the gastrointestinal tissues, as shown in the UK6 and the CD37 groups of animals, regardless of pathology; 2) where large intestinal mucosa was damaged by the pathogenic UK6 strain, HMab IgG leaked into the gut lumen in greater concentrations; and 3) the mild mucosal injury observed was presumably because leaked antibodies protected the gut mucosa from further damage and led to rapid recovery.
